# Antimicrobial Activity of Essential Oils against *Streptococcus mutans* and their Antiproliferative Effects

**DOI:** 10.1155/2012/751435

**Published:** 2012-05-24

**Authors:** Lívia Câmara de Carvalho Galvão, Vivian Fernandes Furletti, Salete Meyre Fernandes Bersan, Marcos Guilherme da Cunha, Ana Lúcia Tasca Góis Ruiz, João Ernesto de Carvalho, Adilson Sartoratto, Vera Lúcia Garcia Rehder, Glyn Mara Figueira, Marta Cristina Teixeira Duarte, Masarahu Ikegaki, Severino Matias de Alencar, Pedro Luiz Rosalen

**Affiliations:** ^1^Department of Pharmacology, Anesthesiology and Therapeutics, Piracicaba Dental School, University of Campinas (UNICAMP), 13414-903 Piracicaba, SP, Brazil; ^2^Research Center for Chemistry, Biology and Agriculture, University of Campinas (UNICAMP), P.O. Box 6171, 13083-970 Campinas, SP, Brazil; ^3^School of Pharmacy and Dentistry, Federal University of Alfenas, 37130-000 Alfenas, MG, Brazil; ^4^Department of Agri-food Industry, Food and Nutrition, Escola Superior de Agricultura “Luiz de Queiroz” (ESALQ), University of São Paulo, P.O. Box 9, 13418-900 Piracicaba, SP, Brazil

## Abstract

This study aimed to evaluate the activity of essential oils (EOs) against *Streptococcus mutans* biofilm by chemically characterizing their fractions responsible for biological and antiproliferative activity. Twenty EO were obtained by hydrodistillation and submitted to the antimicrobial assay (minimum inhibitory (MIC) and bactericidal (MBC) concentrations) against *S. mutans* UA159. Thin-layer chromatography and gas chromatography/mass spectrometry were used for phytochemical analyses. EOs were selected according to predetermined criteria and fractionated using dry column; the resulting fractions were assessed by MIC and MBC, selected as active fractions, and evaluated against *S. mutans* biofilm. Biofilms formed were examined using scanning electron microscopy. Selected EOs and their selected active fractions were evaluated for their antiproliferative activity against keratinocytes and seven human tumor cell lines. MIC and MBC values obtained for EO and their active fractions showed strong antimicrobial activity. Chemical analyses mainly showed the presence of terpenes. The selected active fractions inhibited *S. mutans* biofilm formation (*P* < 0.05) did not affect glycolytic pH drop and were inactive against keratinocytes, normal cell line. In conclusion, EO showed activity at low concentrations, and their selected active fractions were also effective against biofilm formed by *S. mutans* and human tumor cell lines.

## 1. Introduction

Despite the implementation of measures to control and treat dental caries with fluoride, they remain the most prevalent dental disease in many countries [1]. Caries are a multifactorial infectious disease caused by accumulation of biofilm on tooth surface [2]. Manifestations of the disease occur when there is an imbalance between the biofilm and the host due to changes in biofilm matrix pH caused by diet, microorganisms, or salivary flow and their components [3, 4].

*Streptococcus mutans *is considered the most cariogenic of all oral streptococci [5]. *S. mutans* is able to colonize the tooth surface and to produce large amounts of extra and intra-cellular polysaccharides. This microorganism is also highly acidogenic and aciduric, and it metabolizes several salivary glycoproteins, thus being responsible for the initial stage of oral biofilm formation and caries lesions [6].

Several products have been used to control dental caries, such as fluoride, chlorhexidine, and their associations [7]. However, natural products have contributed significantly to the discovery of chemical structures to create new medicaments to be used as innovative therapeutic agents against this prevalent disease [8, 9].

Essential oils (EOs) are important for their detected antimicrobial activity [10–12] including that against *S. mutans* [13]. They are complex, volatile, natural compounds formed by aromatic plants as secondary metabolites [14]. They are known for their bactericidal, virucidal, fungicidal, sedative, anti-inflammatory, analgesic, spasmolytic, and locally anesthetic properties [14]. The presence of complex chemical structures constituted of several groups, such as terpenes and terpenoids, aromatic and aliphatic constituents, all characterized by low molecular weight, may explain their successful bacteriostatic and bactericidal action [14].

Additionally, it was attested that the antimicrobial activity of a natural product, such as EO, is important to evaluate its effects on human normal cell lines and also against human tumor cell lines in order to evidence potential toxicity on human healthy and tumor cell lines [15]. For this reason, it is important that extensive studies involving EO as well as other sources of natural medicines are carried out.

The aim of this study was to evaluate the activity of EO and fractions against planktonic cells of *S. mutans* and also the selected active fractions of EO were chemically characterized and evaluated against mutans biofilm and antiproliferative activity on human cells.

## 2. Materials and Methods

### 2.1. Medicinal Plants

We studied 20 medicinal and aromatic plants (Table 1), which were obtained from the germoplasm bank of the Collection of Medicinal and Aromatic Plants (CPMA) of the Research Center for Chemistry, Biology and Agriculture (CPQBA), University of Campinas (UNICAMP), São Paulo, Brazil (http://www.cpqba.unicamp.br/), and identified by Glyn M. Figueira, curator of CPMA.

The plants were collected from November 2009 to January 2011, during the morning, after the dew point has been reached. The vouchers of each species were deposited in the herbarium of the Institute of Biology, at UNICAMP-UEC, and also registered in the herbarium of CPQBA, receiving identification numbers (CPMA number).

### 2.2. Essential Oil Extraction

 EOs were obtained from 100 g of aerial fresh plant parts by hydrodistillation using a Clevenger-type system, for 3 hours. The aqueous phase was extracted with 50 mL of dichloromethane. Then, the organic layer was separated, dried over anhydrous sodium sulphate (Na_2_SO_4_), and filtered; the solvent was removed by vacuum evaporation at room temperature, resulting in EO. Oil samples were stored at −25°C in sealed glass vials [11].

### 2.3. Fractionation of Essential Oils

 In order to select the EO that should be fractionated, we predetermined some criteria: best antimicrobial activity (MIC < 250 *μ*g/mL), extract yield (>0.5%, except for *Coriandrum sativum* EO), commercial availability, presence of the EO in aerial parts of plants, and easy cultivation. The resulting fractions were also submitted to the antimicrobial assay.

Fractionation was performed using dry column chromatography (cellulose 2 cm × 20 cm) with Si gel 60 (Merck, Darmstadt, Germany) as the stationary phase and dichloromethane as the mobile phase, previously chosen by thin-layer chromatography (TLC), visualized under UV 254 nm, followed by anisaldehyde solution application and drying at 105°C for 5 min. After elution, columns were cut into different parts for each EO, according to polarity and extraction, using dichloromethane. The fractions so obtained were analyzed using TLC and gas chromatography coupled to mass spectrometry (GC-MS) and then bioguided using the antimicrobial assays [16]. All chemical wastes generated during this study were treated according to the Environmental Ethics Committee of UNICAMP (324/2009).

### 2.4. Analyses of the Selected Active Fractions using GC-MS

The chemical composition of each selected active fraction was evaluated using a Hewlett-Packard 6890 gas chromatograph equipped with an HP-5975 mass selective detector and HP-5 capillary column (30 m × 0.25 mm × 0.25 *μ*m). GC-MS was performed using split injection with the injector set at 220°C, the column set at 60°C with a heating ramp of 3°C/min and a final temperature of 240°C, and the MS detector set at 250°C. Helium was used as a carrier gas at 1 mL/min. The GC-MS electron ionization system was set at 70 eV. The quantitative analyses were performed using a Hewlett-Packard 5890 gas chromatograph equipped with a flame ionization detector under the same conditions previously described. A sample of each EO or its selected active fraction was solubilized in ethyl acetate (15 mg/mL) for the analysis. Retention indices (RIs) were determined using injection of hydrocarbon standards and EO samples under the same conditions described above. The oil components were identified by comparison with data described in the literature and the profiles in the NIST 05 mass spectral library [11, 17].

### 2.5. Microorganisms

 For the development of this study, *Streptococcus mutans *UA159 was used.

### 2.6. Antimicrobial Assay

We tested 20 EOs using the antimicrobial assay and selected them according to pre-determined criteria (item 2.3) before being fractionated and continuing the bioguided study.

MIC test was carried out using tissue culture microplates (96 wells) containing 100 *μ*L/well BHI (Brain Heart Infusion, Difco, Franklin Lakes, NJ, USA) medium [18]. The stock solutions of EO and fractions from selected EO (item 2.3) were diluted with propylene glycol (4 mg/mL), transferred to the first well, and serial dilutions were performed to obtain concentrations ranging from 7.81 to 1000 *μ*g/mL. We used 0.12% chlorhexidine (Sigma-Aldrich, St. Louis, MO, USA) as positive control and propylene glycol 6.25% as negative control. The bacterial inoculum (1 × 10^6^ UFC/mL) was added to all wells, and the plates were incubated at 37°C and 5% CO_2_ for 24 hours. MIC was defined as the lowest concentration of EO or fraction from selected EO that inhibited microorganism visible growth indicated by resazurin 0.01% (Sigma-Aldrich, St. Louis, MO, USA) [19].

To determine MBC, an aliquot of each incubated well with concentrations higher than MIC was subcultured on BHI medium supplemented with 5% defibrinated sheep blood using a Whitley Automatic Spiral Plater (Don Whitley Scientific Limited, Shipley, West Yorkshire, UK). MBC was defined as the lowest concentration of EO or fraction that allowed no visible growth on the test medium.

To determine the nature of antibacterial effect of EO and fractions, the MBC : MIC ratio for bacteria was used [20]. When MBC : MIC ratio for *S. mutans* was between 1 : 1 and 2 : 1, the EO or fraction from selected EO was considered bactericidal against this microorganism [20], and when the ratio was higher than 2 : 1, it was considered bacteriostatic.

### 2.7. Action of Selected Active Fractions from Selected EO against *S. mutans* Biofilm

We tested 20 EOs, and those that fulfilled the pre-determined criteria (item 2.3) were selected to be chemically fractionated. The resulting fractions were also tested using the antimicrobial assay and selected according to MIC and MBC results and yields. The selected active fractions were then assessed regarding their action against *S. mutans* biofilm. 

#### 2.7.1. Inhibition of *S. mutans* Biofilm Growth

In order to evaluate the antimicrobial activity of EO selected active fractions against the formation of *S. mutans* biofilm, the samples were placed, at different concentrations (7.81–1000 *μ*g/mL), in the wells of sterile polystyrene U-bottom microtiter plates, previously treated with saliva (the use of human saliva in this study was approved by the Research Ethics Committee of the Piracicaba Dental School, State University of Campinas (UNICAMP) (Approval 087/2011)) [21].* S. mutans* cells (1.0 × 10^7^ cells/mL in BHI medium) were added to wells containing BHI medium with 2% sucrose and the samples were incubated at 37°C for 18 hours. Biofilm growth was revealed and quantified using the crystal violet staining method and measuring absorbance at 575 nm [11, 22].

After 18 hours of incubation, the spent medium was aspirated, nonadhered cells were removed, the wells were washed three times with sterile distilled water, and the plates were dried for 45 min before carrying out biofilm quantification [22].

#### 2.7.2. Glycolytic pH-Drop Assay

The effect of EO selected active fractions against *S. mutans *biofilm was measured using the standard glycolytic pH-drop assay [23]. Biofilm growth was carried out as previously described (item 2.7.1), in sterile polystyrene U-bottom microtiter plates without fractions. The biofilms so obtained were washed twice with 0.9% NaCl solution and salt solution (50 mM KCl + 1.0 mM MgCl_2_), containing EO selected active fractions at different concentrations (1000, 500, and 250 *μ*g/mL), and vehicle (25% propylene glycol, v/v) was added. The pH was adjusted to 7.2 with 0.1 M KOH solution, and glucose was added to a final concentration of 1%, and pH-drop was assessed using Orion pH glass electrode attached to Orion 290 A^+^ pHmeter (Orion Scientific, Houston, TX, USA) for 90 min.

### 2.8. Scanning Electron Microscopy (SEM)

 In order to evaluate *S. mutans* integrity using SEM, biofilms were first developed in Lab-Tek chambered coverglass (Nunc, Naperville, IL, USA), as described previously (item 2.7.1), were treated with vehicle (6.12% propylene glycol) or had their active fractions selected at concentrations able to inhibit more than 90% of *S. mutans* biofilm formation. Samples were fixed in 4% glutaraldehyde (v/v) in phosphate-buffered saline (PBS) at room temperature for 12–24 hours. After this procedure, the biofilms were dehydrated through a graded series of ethanol (50% to 100%), dried to a critical point, coated with gold, and observed using a scanning electron microscope JEOL JSM5600LV (JEOL Ltd., Tokyo, Japan) [11, 24].

### 2.9. Antiproliferative Assay

The *in vitro a*ntiproliferative assay [25] was performed in the present study using a human keratinocyte (HaCat) cell line, kindly donated by Dr. Ricardo Della Coletta (FOP, UNICAMP, Brazil), and seven human tumor cell lines (U251 (glioma), MCF-7 (breast), NCI-ADR/RES (ovarian expressing phenotype multiple drugs resistance), 786-0 (renal), NCI-H460 (lung, nonsmall cells), PC-3 (prostate), and OVCAR-03 (ovarian), kindly provided by M. A. Frederick (National Cancer Institute, USA). Stock and experimental cultures were grown in medium containing 5 mL RPMI-1640 (Gibco-BRL, Grand Island, NY, USA) supplemented with 5% fetal bovine serum (Gibco-BRL, Grand Island, NY, USA). A penicilline-streptomicine mixture (1000 U/mL : 1000 mg/mL, 1 mL/L RPMI) was added to experimental cultures. Cells in 96-well plates (100 *μ*L cells/well) were exposed to each EO and selected active fractions in dimethyl sulfoxide (DMSO, Sigma-Aldrich, St. Louis, MO, USA) (0.25, 2.5, 25, and 250 *μ*g/mL) at 37°C and 5% CO_2_ for 48 hours. Final DMSO concentration did not affect cell viability. Before (T_0_ plate) and after sample addition (T_1_ plates), cells were fixed with 50% trichloroacetic acid and cell proliferation was determined by spectrophotometric quantification (540 nm) of cellular protein content using sulforhodamine B assay. Using the concentration-response curve for each cell line, the total growth inhibition (TGI) was determined by nonlinear regression analysis using the software Origin 8.0 (OriginLab Corporation, Northampton, MA, USA) [26, 27].

### 2.10. Statistical Analysis

An exploratory data analysis was performed to determine the most appropriate statistical test. Inhibition of biofilm growth, and glycolytic pH-drop data were compared using the nonparametric Kruskal-Wallis test. *P* value < 0.05 was considered statistically significant. Triplicates from at least three separated experiments were conducted in each assay.

## 3. Results

### 3.1. Essential Oils and Fraction Yields

The EO yields, expressed in relation to dry weight of plant material (%, w/w), are shown in Table 1.

According to pre-determined criteria (item 2.3), four EOs were selected to be fractionated using dry column as follows: *A. gratissima, B. dracunculifolia, C. sativum*, and* L. sidoides*.

The yields of the fractions from selected EO were expressed as a function of the respective EO yield (%, w/w) and are shown in Table 2. The yields of *A. gratissima *fractions ranged from 14.4% to 29%, *B. dracunculifolia *from 20.1% to 30.6%, *C. sativum* from 4.9% to 30.9%, and *L. sidoides* from 1.7% to 33.3%.

### 3.2. Antimicrobial Activity

MIC and MBC values for all tested EO are shown in Table 1. MIC values ranged from 31.2 to 500 *μ*g/mL, and MBC values ranged from 62.5 to 1000 *μ*g/mL. The highest activities were observed for *A. gratissima *and *A. triphylla *(125–250 *μ*g/mL)*, B. dracunculifolia*, *L. sidoides*, *M. glomerata*, *S. guianenses*, *S. aromaticum *(62.5–125 *μ*g/mL), and* C. sativum *(31.2–62.5 *μ*g/mL).

Based on pre-determined criteria (item 2.3), four EOs (*A. gratissima, B. dracunculifolia, C. sativum*, and* L. sidoides*) were selected to be fractionated. MIC and MBC values of fractions from selected EO are shown in Table 2. MIC values obtained for all fractions ranged from 15.6 to 500 *μ*g/mL, and MBC values ranged from 31.2 to 1000 *μ*g/mL. The highest activities were observed for the fractions Ag_4_ (31.2–62.5 *μ*g/mL), Bd_2_ (15.6–31.2 *μ*g/mL), Cs_4 _(15.6–31.2 *μ*g/mL), and Ls_3_ (62.5–125 *μ*g/mL).

The MBC : MIC ratio (Table 1) showed that most EOs are bactericidal, except for *B. dracunculifolia, E. florida*, and* S. aromaticum*, which are considered bacteriostatic against *S. mutans*. Among the selected EO chosen to be fractionated, only that obtained from *B. dracunculifolia* was bacteriostatic. Most fractions from selected EO were bactericidal, except for Ag_4_, Cs_1_, Ls_2_, and Bd_2_, considered bacteriostatic against *S. mutans* (Table 2). Based on yield and antimicrobial activity, Ag_4_, Bd_2_, Cs_4_, and Ls_3_ fractions were selected for further evaluations.

### 3.3. Selected Active Fractions Activity against *S mutans* Biofilm

**Figure 1:**
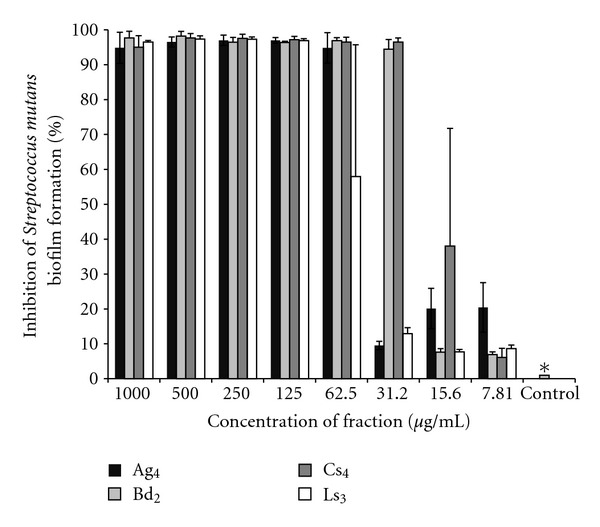
Influence of selected active fractions Ag_4_, Bd_2_, Cs_4_, and Ls_3_ from selected essential oils at different concentrations against *Streptococcus mutans* biofilm formation. All fractions tested were significantly different from the vehicle at all concentrations tested. Kruskal-Wallis test (*P* < 0.05).

Figure 1 shows the development of *S. mutans* biofilm inhibiton after treatment with selected active fractions. Their growth was measured by optic density at 575 nm. The result showed that the selected active fractions tested at different concentrations were significantly different (*P* < 0.05) from the vehicle. Moreover, Cs_4_ and Bd_2_ fractions presented a better performance since they inhibited more than 90% of biofilm formation at lower concentrations (31.2 *μ*g/mL).

### 3.4. pH-Drop Assay

 The influence of selected active fractions from EO on glycolytic pH-drop of *S. mutans* biofilm formation in the presence of excess glucose was not significant (*P* > 0.05) for all fractions tested (Ag_4_, Bd_2_, Cs_4_, and Ls_3_).

### 3.5. Chemical Characterization of Fractions Constituents

The chemical composition of the selected EO and the selected active fractions is shown in Table 3.

The analyses of EO and fractions indicated the presence of volatile compounds, mainly mono- and sesquiterpenes.

We identified 28 compounds in the EO of* A. gratissima*, representing 92.73% of the EO, 25 compounds in the EO of* B. dracunculifolia*, representing 93.45% of the EO, 15 compounds in the EO of* C. sativum*, representing 91.93% of the EO, and four compounds in the EO of* L. sidoides*, representing 100% of the EO. We also identified 19 compounds in fraction Ag_4_, representing 94.6% of the fraction, 10 compounds in fraction Bd_2_, representing 83.06% of the fraction, nine compounds in fraction Cs_4_, representing 89.71% of the fraction, and five compounds in fraction Ls_3_, representing 99.7% of the fraction.

The major compounds identified in each selected EO were: trans- and cis-pinocamphone, beta-pinene, and guaiol in* A. gratissima*; trans-nerolidol and spathulenol in* B. dracunculifolia*; 2-decen-1-ol and 1-decanol in* C. sativum*; and thymol in *L. sidoides*. The major compounds identified in each selected fractions were trans- and cis-pinocamphone and guaiol in Ag_3_; trans-nerolidol, spathulenol, and ethyl ester benzenepropanoic in Bd_2_; 2-decen-1-ol and 1-decanol in Cs_4_; thymol in Ls_3_.

### 3.6. Scanning Electron Microscopy (SEM)

**Figure 2:**
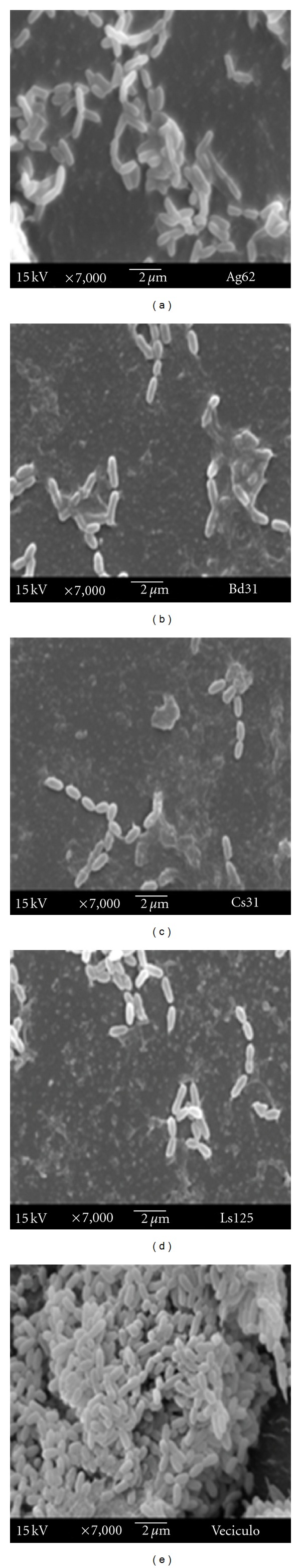
Scanning electron microscopy of *Streptococcus mutans* biofilms treated with the selected active fractions from selected essential oils and the vehicle. Images a, b, c, and d show the reduction of biofilm formation after treatment with Ag_4_, Bd_2_, Cs_4_, and Ls_3_ fractions, respectively, compared with the treatment with the vehicle (image (e)) (magnification of 7000x).

The effect of selected active fractions against *S. mutans* biofilm formation was evaluated by SEM.  Figure 2 shows a reduction in biofilm formation. Biofilms were first developed as described previously (Section 2.7.1), were treated with vehicle, or had their active fractions selected at concentrations able to inhibit more than 90% of *S. mutans* biofilm formation (Ag_4_ at 62.5 *μ*g/mL, Bd_2_ and Cs_4_ at 31.2 *μ*g/mL, and Ls_3_ at 125 *μ*g/mL).

### 3.7. Antiproliferative Assay

Most EOs and their selected active fractions did not present activity against the human normal cell line evaluated in this study or presented high concentrations to totally inhibit its growth. TGI values are shown in Table 4.

Among the EO evaluated, *B. dracunculifolia* and *C. sativum* were the most active inhibitors of human tumor cell lines growth, presenting selectivity for U251 (TGI = 38.2 *μ*g/mL and TGI = 8.3 *μ*g/mL, resp.) and OVCAR-3 (TGI < 0.25 *μ*g/mL for both). On the other hand, *A. gratissima* and *L. sidoides* displayed the lowest activity, both presenting selectivity for OVCAR-3 (TGI < 0.25 *μ*g/mL for both) and *L. sidoides* for PC-3 (TGI = 26.7 *μ*g/mL). The reference compound, doxorubicin, presented antiproliferative activity against all cell lines, except for kidney (Table 4).

Table 4 also shows the activity of selected active fractions. Ag_4_ and Ls_3_ fractions presented better results than *A. gratissima* and *L. sidoides* EO, respectively, since these fractions were not active against human normal cell lines (TGI > 250 *μ*g/mL) and showed lower TGI values, being selective for 786-0 (TGI = 5.9 *μ*g/mL and TGI = 26.7 *μ*g/mL, resp.). Cs_4_ fraction had better results than *C. sativum* EO only against NCI-ADR/RES (TGI = 13.1 *μ*g/mL and TGI = 90 *μ*g/mL, resp.). Bd_2_ displayed a better performance than *B. dracunculifolia* EO against NCI-ADR/RES (TGI = 10.5 *μ*g/mL and TGI = 59.2 *μ*g/mL, resp.), 786-0 (TGI = 47.1 *μ*g/mL and TGI = 49.5 *μ*g/mL, resp.), and NCI-H460 (TGI = 76.8 *μ*g/mL and TGI = 87.6 *μ*g/mL, resp.).

## 4. Discussion

The activity of natural products, especially EO, against microorganisms has been recently confirmed by several studies focusing on antimicrobial activity of EO against planktonic cells. However, bacteria growing in biofilms exhibit a specific phenotype and are often, but not always, more resistant to antimicrobial agents than their planktonic counterparts [10, 11]. Thus, it is important to search for natural products that have antibiofilm properties and antimicrobial activity against oral pathogens [28].

This study aimed to evaluate the activity of EO and their fractions against planktonic cells of *S. mutans,* and the active fractions were evaluated against biofilm formed by *S. mutans*. Also, EO and their active fractions were chemically characterized and their activity against human normal and tumor cell lines proliferation were determined.

The antimicrobial assay revealed low MIC values for almost all 20 EOs and 15 fractions from the selected EO tested. EO and the selected active fractions presented strong activity against *S. mutans*, since natural products are considered strong inhibitors of microbial activity, when MIC values are lower than 500 *μ*g/mL [29].

These results demonstrate that the EO studied and especially those selected (*A. gratissima, B. dracunculifolia, C. sativum*, and* L. sidoides*) have potential for bioprospection of new active biomolecules. The fractionation process adopted showed good results, since the fractions obtained were more active than the original EO (Table 2). This bioguided study is a model for bioprospecting new drugs [30], and it can be considered successful since we found active fractions presenting higher activity than their respective EO.

Most EO and fractions studied showed MBC : MIC ratio that enables them to be classified as bactericidal compounds. This could be explained by their hydrophobicity, an important characteristic that exists in EO and their fractions [31] and may allow them to partition the lipids of the bacterial cell membrane, turning them more permeable and leading to leakage of ions and other cell constituents [32, 33]. On the other hand, *B. dracunculifolia *EO and its selected active fraction (Bd_2_) present compounds that could be capable of infiltrating the cell and interact with cellular metabolic mechanisms [34], demonstrating their bacteriostatic effect. Nevertheless, despite presenting bactericidal or bacteriostatic effect, the selected EO proved to be active against both *S. mutans* planktonic cells and biofilm, demonstrating the effectiveness of the substances present in these EO, since it is difficult to disrupt *S. mutans* biofilm [35].

The selected active fractions were also tested against *S. mutans* biofilm, and they were able to disrupt its formation at all tested concentrations. This disruption was observed using SEM, which showed the change the selected active fractions caused in the structure of *S. mutans *biofilm.

At the concentrations tested, it was possible to observe huge failures in *S. mutans* biofilm surface treated with the active fractions when compared with the treatment with the vehicle, which presented a more homogeneous biofilm surface. These changes were also observed in another study that tested the action of *C. sativum* and its bioactive fraction against *Candida albicans* [11]. Moreover, the simple conformational change in biofilm, caused by the action of the selected active fractions, could make it more susceptible and less virulent [4].

However, when the selected active fractions were tested in order to evaluate their ability to reduce *S. mutans* acid production, no significant results were observed (*P* > 0.05). Therefore, the selected active fractions could not act on this important virulence factor of *S. mutans*, different from the findings of another work with *B. dracunculifolia* extracts, which showed significant reduction in production of acid by this microorganism [36]. The difference between *B. dracunculifolia* EO and the active extracts from this plant may be attributed to the extraction method, which results in different compound mixtures with different mechanisms of action [37].

It is known that EOs are composed of numerous different chemical compounds, and their antimicrobial activity might be attributed to several different mechanisms, which could explain the variations in their mode of action [38].

The present data suggest the occurrence of a separation during the fractionation process of the selected EO in such a way that the selected active fractions presented higher amounts of bioactive compounds than their respective EO. The main biologically active compounds found in the selected active fractions were thymol, carvacrol, 2-decen-1-ol, trans-nerolidol, spathulenol, ethyl ester benzenepropanoic, trans-pinocamphone, cis-pinocamphone, and guaiol. These compounds have been extensively described in the literature for their effect on microorganisms [39, 40].

Both forms of trans- and cis-pinocamphone are major constituents of Ag_4_ fraction and were also found in *Hyssopus officinalis *L. EO [41]. These compounds are responsible for the antibacterial, antifungal, and antioxidant activities of *H. officinalis *EO, demonstrating that they pass through the cell wall and the plasma membrane, disrupting their structure [41]. The bactericidal activity of Ag_4_ fraction observed in the present study may be a consequence of this mode of action.

Trans-nerolidol and spathulenol, two compounds present in Bd_2_ fraction, have been considered active against unknown Gram-positive and Gram-negative bacteria [13]. Although spathulenol shows activity against *S. mutans*, its mechanism of action still remains unknown [13].

 Other studies showed that certain alcohols, such as 2-decen-1-ol, have higher antimicrobial activity than aldehydes against *Candida *ssp. [11, 16]. These alcohols were found in Cs_4_ fraction and may be responsible for the action against *S. mutans* biofilm. Furthermore, considering the mode of action of *C. sativum* EO, it seems to result in bacterial cell permeabilization, leading to the impairment of other cell functions, such as membrane potential, respiratory activity, or efflux pump activity [42].

Thymol is an optic isomer of carvacrol, and both substances seem to make bacterial membrane more permeable [43]. In our study, both were found in Ls_3_ fraction as its major components. Previous studies have shown that these compounds present antimicrobial activity against fungi and bacteria [44], including species of the genus *Streptococcus *[12].

After determining the antimicrobial activity of a natural product, it is important to verify if it also exhibits antiproliferative activity, mainly after its fractionation, a procedure that may concentrate toxic compounds in the fractions that present biological activity.

Based on TGI values, the selected EO and selected active fractions could be classified as inactive (TGI > 50 *μ*g/mL), weakly active (15 *μ*g/mL < TGI < 50 *μ*g/mL), moderately active (6.25 *μ*g/mL < TGI < 15 *μ*g/mL), and strongly active (TGI < 6.25 *μ*g/mL) [45]. The absence of activity was clearly observed in this study since all selected EO and selected active fractions were inactive against the human normal cell line tested.

All EOs tested were selective against the ovarian tumor cell line, showing potent activity. Ag_4_ showed potent activity against the kidney tumor cell line, and Bd_2_ and Cs_4_ fractions showed only moderate activity against the ovarian tumor cell line. These results show the specificity of these EO and their fractions against some tumor cell lines, an important and desired characteristic for potential new chemotherapic drugs [15].

It is known that EO compounds, such as monoterpenes, have shown effects on mevalonate metabolism, linked to the maintenance of cell membrane, which could contribute to terpene tumor suppressive action [46]. Thereby, the presence of monoterpenes in the selected active fractions of our study may explain their antiproliferative actions against some tumor cell lines [47]; however, more studies are required to find the compounds of EO responsible for their anticancer activity, since little is known about essential oils and their antiproliferative activity.

## 5. Conclusion

The results of the present study indicate that all EO and fractions tested showed good antimicrobial activity, but only those showing activity at low concentrations were taken into consideration and fractionated for bioprospection of new agents against *S. mutans*. Among these fractions, the selected active fractions were able to disrupt *S. mutans *biofilm formation, did not inhibit normal cell line growth, and were more specific against human tumor cell lines. These features enable them to be tested in further studies and help the discovery of new bioactive molecules.

## Figures and Tables

**Table 1:** Medicinal and aromatic plants from the germplasm bank of the CPMA/CPQBA/UNICAMP selected for this study with their yield, MIC and MBC values, and MBC : MIC ratio.

Medicinal species	Family	Popular name	Source	CPMA number	Voucher number^1^	Yield (%)	MIC (*μ*g/mL)	MBC (*μ*g/mL)	MBC : MIC ratio^2^	Popular use
*Aloysia gratissima *(Gillies & Hook)	Verbenaceae	Brazilian lavender	Leaf	714	UEC 121.393	1.1	125–250	250–500	2 : 1	Digestive; antispasmodic
*Aloysia triphylla *(L'Hér.) Britton	Verbenaceae	Aloisia	Leaf	274/700	UEC 121.412	0.3	125–250	125–250	1 : 1	Sedative; antispasmodic
*Alpinia speciosa *(Pers.) Burtt & Smith	Zingiberaceae	Colony	Root	447	UEC 145.185	0.2	125–250	250–500	2 : 1	Antimicrobial
* Baccharis dracunculifolia DC*	Asteraceae	Broom weed	Leaf	1841	—	0.8	62.5–125	250–500	4 : 1	Tonic; eupeptic, antipyretic
*Cinnamomum zeylanicum* Blume	Lauraceae	Cinnamon	Leaf	455	IAC 19624	0.2	250–500	500–1000	2 : 1	Carminative; antispasmodic
*Coriandrum sativum *L.	Apiaceae	Coriander	Leaf	664	—	0.3	31.2–62.5	62.5–125	2 : 1	Antimicrobial; antifungal
*Cymbopogon citratus *(DC) Stapf.	Poaceae	Lemon grass	Leaf	503	UEC 85.210	1.1	125–250	250–500	2 : 1	Sedative; analgesic; anticough
*Cymbopogon martini* (Roxb.) J. F. Watson	Poaceae	Palmarosa	Leaf	354	UEC 127.115	0.6	125–250	250–500	2 : 1	Antiseptic; antifungal
*Cymbopogon winterianus *Jowitt	Poaceae	Lemon verbena	Leaf	712	UEC 121.414	1.5	125–250	250–500	2 : 1	Repellent, insecticide
*Cyperus articulatus *Vahl	Cyperaceae	Priprioca	Bulbs	222	UEC 121.396	0.5	125–250	250–500	2 : 1	Anti-inflammatory
*Elyonurus muticus *Spreng	Poaceae	Agripalma	Leaf	1701	UEC 20.580	0.6	125–250	125–250	1 : 1	Antibacterial
*Eugenia florida *DC.	Myrtaceae	Guamirim-cereja	Leaf	1685	IAC 49207	0.3	125–250	500–1000	4 : 1	Anti-inflammatory
*Eugenia uniflora L.*	Myrtaceae	Pitanga	Leaf	1816	—	0.7	125–250	250–500	2 : 1	Antihypertensive; diuretic
*Lippia alba *(Mill.) N.E. Brown	Verbenaceae	False lemon balm	Leaf	467/509	UEC 121.413	0.3	125–250	250–500	2 : 1	Treatment of migraines
*Lippia sidoides *Cham.	Verbenaceae	Rosemary	Leaf	398/399	—	4.7	62.5–125	125–250	2 : 1	Bactericide; fungicide
*Mentha piperita *L.	Lamiaceae	Mint	Leaf	560	UEC 127.110	2.2	250–500	250–500	1 : 1	Antifungal; antibacterial
*Mikania glomerata *Spreng.	Asteraceae	Guaco	Leaf	766	UEC 102.047	0.4	62.5–125	125–250	2 : 1	Anti-inflammatory; bronchodilator
*Siparuna guianenses *Aubl.	Monimiaceae	Wild lemon	Leaf	2025	—	0.3	62.5–125	125–250	2 : 1	Tranquilizer; diuretic
*Syzygium aromaticum* (L.) Merr. & L. M. Perry	Myrtaceae	Cloves	Leaf	455	IAC 19624	0.5	62.5–125	250–500	4 : 1	Seasoning; antibacterial
*Ziziphus joazeiro *Mart.	Rhamnaceae	Joazeiro fruit	Leaf	2119	—	0.5	250–500	500–1000	2 : 1	Astringent; anti-inflammatory

^1^A voucher herbarium specimen is a pressed plant sample deposited for future reference. Vouchers deposited at UEC herbarium (http://www.ib.unicamp.br/herbario/) at Biology Institute (IB) of UNICAMP, SP, Brazil. (—) Species with no voucher number registered. ^2^The EOs were considered bactericidal when the MBC : MIC ratio was between 1 : 1 to 2 : 1, and bacteriostatic if this ratio was higher than 2 : 1.

**Table 2:** Selected EO and their fractions with yield results, MIC and MBC values, and MBC : MIC ratio.

Essential oil			Fraction				
Identification	MIC (*μ*g/mL)	MBC (*μ*g/mL)	Identification	Yield (%)	MIC (*μ*g/mL)	MBC (*μ*g/mL)	MBC : MIC ratio^1^
*Aloysia gratissima *(Ag)	125–250	250–500	Ag_1_	28.9	250–500	500–1000	2 : 1
			Ag_2_	17.9	250–500	500–1000	2 : 1
			Ag_3_	20.1	62.5–125	500–1000	8 : 1
			**Ag_4_** ^2^	**14.4**	**31.2–62.5**	**62.5–125**	**2 : 1**
*Baccharis dracunculifolia *(Bd)	62.5–125	250–500	Bd_1_	30.5	250–500	500–1000	2 : 1
			**Bd_2_**	**22.1**	**15.6–31.2**	**125–250**	**8 : 1**
*Coriandrum sativum* (Cs)	31.2–62.5	62.5–125	Cs_1_	6.6	125–250	500–1000	4 : 1
			Cs_2_	4.9	125–250	250–500	2 : 1
			Cs_3_	12.7	15.6–31.2	31.2–62.5	2 : 1
			**Cs_4_**	**30.9**	**15.6–31.2**	**31.2–62.5**	**1 : 1**
*Lippia sidoides* (Ls)	62.5–125	125–250	Ls_1_	13.6	250–500	500–1000	2 : 1
			Ls_2_	33.3	62.5–125	250–500	4 : 1
			**Ls_3_**	**26**	**62.5–125**	**125–250**	**2 : 1**
			Ls_4_	6.1	62.5–125	125–250	2 : 1
			Ls_5_	1.7	62.5–125	125–250	2 : 1

^1^The fractions from selected EO were considered bactericidal when the MBC : MIC ratio was between 1 : 1 to 2 : 1, and bacteriostatic if this ratio was higher than 2 : 1. ^2^The fractions in bold font were selected as active fractions and evaluated against *S. mutans* biofilm and for their antiproliferative action. The subscript numbers of the fractions represent the numbers of parts obtained using the dry column fractionation.

**Table 3:** Major compounds of the selected active fractions from essential oils with their retention time (Rt), retention index (RI), and relative percentage.

Rt (min)	RI	Compound				Relative percentage^1^				
			Ag EO	Ag_4_	Bd EO	Bd_2_	Cs EO	Cs_4_	Ls EO	Ls_3_
4.02	899	Cyclohexanone	—	—	—	—	—	—	6.5	—
4.22	850	3-hexen-1-ol	—	—	—	0.8	3.6	5.1	—	—
5.87	977	Beta-pinene	12.0	—	—	—	—	—	—	—
7.2	1024	p-cymene	—		—	—	—	—	17.3	—
13.08	1140	Trans-pinocarveol	—	4.9	—	—	—	—	—	—
**14.09**	**1165**	**Trans-pinocamphone**	**16.0**	**36.7**	**—**	**—**	**—**	**—**	**—**	**—**
**14.61**	**1177**	**Cis-pinocamphone**	**6.0**	**17.0**	**—**	**—**		**—**	**—**	**—**
**16.7**	**1274**	**2-decen-1-ol <E>**	**—**	**—**	**—**	**—**	**23.6**	**26.9**	**—**	**—**
**16.86**	**1277**	**1-decanol**	**—**	**—**	**—**	**—**	**33.9**	**35.4**	**—**	**—**
17.76	1299	Trans-pinocarvyl acetate	8.2	—	—	—	—	—	—	—
**19.74**	**1300**	**Thymol**	**—**	**—**	**—**	**—**	**—**		**65.8**	**97.8**
19.95	1303	Carvacrol	—	—	—	—	—	—	—	0.6
21.84	1349	Ethyl ester benzenepropanoic	—	—	—	11.7	—	—	—	—
22.57	1416	Trans-caryophyllene	7.2	—	10.7	—	—	—	10.5	—
24.86	1473	2-dodecen-1-ol	—	—	—	—	13.1	14.5	—	—
25.04	14.78	Germacrene D	—	—	4.9	—	—	—	—	—
25.66	1493	Bicyclogermacrene	4.2	—	6.8	—	—	—	—	—
27.97	1553	M^2^ = 204	6.4	—	—	—	—		—	—
**30.59**	**1566**	**Trans-nerolidol**	**—**	**—**	**31.7**	**52.2**	**—**	**—**	**—**	**—**
**31.05**	**1578**	**Spathulenol**	**—**	**—**	**13.6**	**11.5**	**—**	**—**	**—**	**—**
31.23	1582	Caryophyllene oxide	6.4	7.0	—	6.3	—	—	—	0.7
31.9	1600	Guaiol	8.5	12.7	—	—	—	—	—	—
33.44	1641	Epi alpha cadinol	—	—	—	3.1	—	—	—	—
32.47	1674	2-tetradecen-1-ol <E>	—	—	—	—	5.5	5.2	—	—
34.40	1668	Bulnesol	—	3.5	—	—	—	—	—	—

^1^The selected active fractions Ag_4_, Bd_2_, Cs_4_, and Ls_3_ had their actions against *S. mutans* biofilm and their antiproliferative activity evaluated. Ag EO, Bd EO, Cs EO, and Ls EO correspond to the following essential oils: *Aloysia gratissima, Baccharis dracunculifolia, Coriandrum sativum*, and *Lippia sidoides*, respectively. Only the compounds with relative percentage above 3% are listed. ^2 ^M: molecular weight of a nonidentified compound.

**Table 4:** Total growth inhibition (TGI) of selected essential oils and their selected active fractions tested against normal human cell and tumor cell lines.

Cell line		TGI (*μ*g/mL)^1^							
	Ag EO	Ag_4_	Bd EO	Bd_2_	Cs EO	Cs_4_	Ls EO	Ls_3_	Dox
Glioma (U251)	>250	55.6	**38.2**	51.4	**8.3**	61.5	>250	94.9	0.92
Breast (MCF-7)	>250	45.2	46.0	67.7	**13.6**	111.6	>250	56.6	3.3
Ovarian (NCI-ADR/RES)	>250	50.6	59.2	**10.5**	90.0	**13.1**	>250	112.3	1.6
Kidney (786-0)	>250	**5.9**	49.5	47.1	**29.8**	72.1	>250	**26.7**	>250
Lung (NCI-H460)	>250	42.7	87.6	76.8	105.0	110.3	>250	79.8	4.9
Prostate (PC-3)	99.9	>250	>250	>250	118.1	141.9	**26.7**	>250	11.7
Ovarian (OVCAR-3)	**<0.25**	47.6	**<0.25**	58.0	**<0.25**	73.7	**<0.25**	60.4	7.6
Keratinocytes (HaCaT)	>250	>250	92.3	95.7	129.4	145.6	>250	>250	2.3

^1^Data result from three replicates per treatment in two independent tests at 25°C for 48 hours. Ag EO, Bd EO, Cs EO, and Ls EO correspond to the following essential oils: *Aloysia gratissima, Baccharis dracunculifolia, Coriandrum sativum*, and *Lippia sidoides*, respectively. Ag_4_, Bd_2_, Cs_4_, and Ls_3_ are the selected active fractions evaluated. Dox: doxorubicin (positive control).
